# Assessing Causal Mechanistic Interactions: A Peril Ratio Index of Synergy Based on Multiplicativity

**DOI:** 10.1371/journal.pone.0067424

**Published:** 2013-06-24

**Authors:** Wen-Chung Lee

**Affiliations:** 1 Research Center for Genes, Environment and Human Health, College of Public Health, National Taiwan University, Taipei, Taiwan; 2 Institute of Epidemiology and Preventive Medicine, College of Public Health, National Taiwan University, Taipei, Taiwan; University of Texas School of Public Health, United States of America

## Abstract

The assessments of interactions in epidemiology have traditionally been based on risk-ratio, odds-ratio or rate-ratio multiplicativity. However, many epidemiologists fail to recognize that this is mainly for statistical conveniences and often will misinterpret a statistically significant interaction as a genuine mechanistic interaction. The author adopts an alternative metric system for risk, the ‘peril’. A peril is an exponentiated cumulative rate, or simply, the inverse of a survival (risk complement) or one plus an odds. The author proposes a new index based on multiplicativity of peril ratios, the ‘peril ratio index of synergy based on multiplicativity’ (PRISM). Under the assumption of no redundancy, PRISM can be used to assess synergisms in sufficient cause sense, i.e., causal co-actions or causal mechanistic interactions. It has a less stringent threshold to detect a synergy as compared to a previous index of ‘relative excess risk due to interaction’. Using the new PRISM criterion, many situations in which there is not evidence of interaction judged by the traditional indices are in fact corresponding to *bona fide* positive or negative synergisms.

## Introduction

Epidemiologists are often troubled by how to make sense of the joint exposure effects of two factors on the risk of a disease. For example, is the combined effect of tobacco smoking and asbestos exposure greater (or smaller) than what would be expected based on their individual separate effects? And, if the combined effect indeed deviates from its expectation, does it imply a mechanistic interaction between the two exposures? These apparently simple questions prove difficult to answer. First, we need a suitable scale with which to measure an effect. But which scale to use, a ratio scale (e.g., risk ratio, odds ratio and rate ratio) or a difference scale (e.g., risk difference, odds difference and rate difference)? Second, we need to define a reasonable expectation of the combined effect, against which to define an interaction. But what type of interactions: a multiplicative interaction or an additive interaction?

The assessments of interactions have traditionally been based on risk-ratio, odds-ratio or rate-ratio multiplicativity, such as using the ‘synergistic index of multiplicativity’ (SIM) [1]. No multiplicative interaction (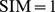) corresponds to risk-ratio, odds-ratio or rate-ratio homogeneity across strata in a stratified analysis, or to absence of cross-product terms in a multiplicative model, such as logistic, Poisson or Cox regression. The use of multiplicative models is so dominating in epidemiology that a significant multiplicative interaction is often mistaken as a genuine mechanistic interaction.

Recently, an index of risk-ratio additivity, the ‘relative excess risk due to interaction’ (RERI) [2], has received much attention. (For two dichotomous exposures 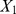 and 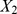, 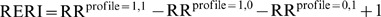, where 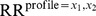 is the risk ratio comparing the disease risks between those with exposure profile of 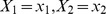 and those with 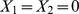.) The index can assess synergisms in sufficient cause sense, i.e., causal co-actions or causal mechanistic interactions [3]–[6]. A sufficient cause [7] contains a combination of component causes. There may be many classes of sufficient causes for a disease. Any class with all its components completed is sufficient to cause the disease. For the aforementioned example, we may speak of synergisms if there are some lung cancer patients who had developed the disease because of the completions of the classes of sufficient causes containing *both* tobacco *and* asbestos as their components.

As the RERI above is based on ‘risks’, it necessarily entails follow-up of a population for a certain period, say, from time 0 to time 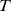. The dependency on an arbitrary time point 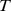 is rather undesirable. First, it is possible that two researchers using different 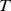
s will reach different conclusions regarding causal mechanistic interactions. And second, when 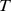 tends to infinity, 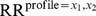 will tend to one for each and every 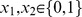, and RERI will tend to zero (perfect risk-ratio additivity). This thus masks any possible synergism between 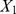 and 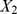
!.

In this paper, I turn to ‘rates’ instead. Because all rates are defined the same way with their 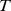
s being made to be infinitesimally small, an index of rate does not have the above 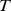 dependency problem. An alternative metric of risk is then used: the exponentiated cumulative rate, which I refer to as the ‘peril’. I will show that the synergy index based on multiplicativity of peril ratios, the ‘peril ratio index of synergy based on multiplicativity’ (PRISM), can be used to assess synergisms in sufficient cause sense: perfect multiplicativity (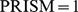) implying no synergism, super-multiplicativity (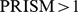), positive synergisms, and sub-multiplicativity (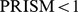), negative synergisms, respectively. I will also show that PRISM has a less stringent threshold to detect a synergy as compared to RERI, and that many situations in which there is not evidence of interaction judged by the traditional indices of SIM and RERI are in fact corresponding to *bona fide* positive or negative synergisms.

## Methods

Consider the relation of two dichotomous exposures and a disease in a follow-up of a population in a certain time interval, (0, 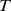). I assume that the exposure status is time-invariant and the follow-up is 100% complete (without loss to follow up and competing death). For people in the population with a risk factor profile of 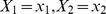, let 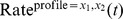 denote the (instantaneous) disease rate at follow-up time 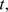 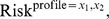 the cumulative disease risk (probability) in (0, 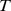), 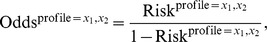 the cumulative disease odds in (0, 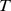), and 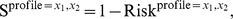 the survival probability at 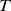. I assume that there is no confounding, selection bias or measurement error in this cohort study, such that the associations between the two exposures and the disease should reflect the genuine causal effects of the exposures on the disease.

**Figure 1:**
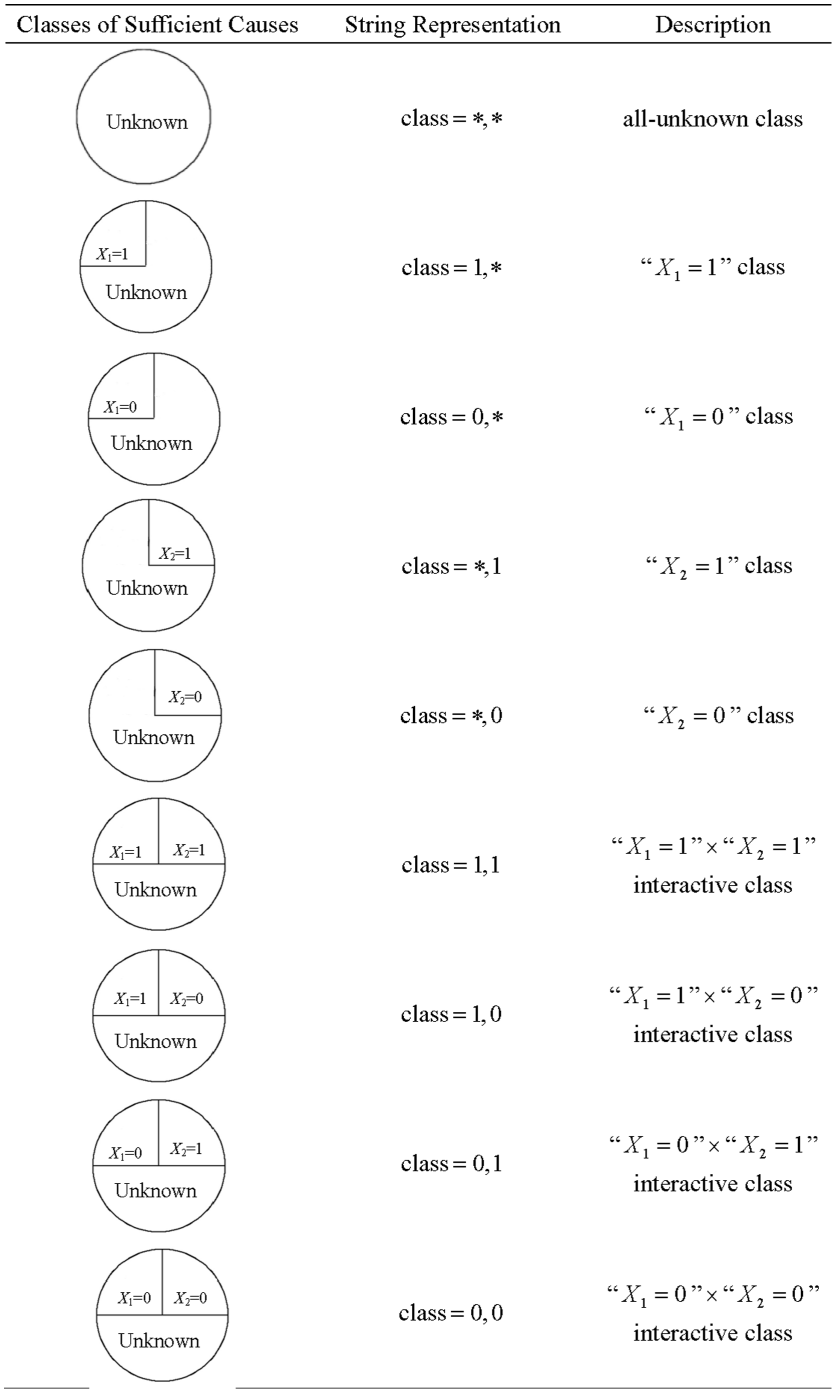
The total 9 classes of sufficient causes for 2 dichotomous exposures.

For two dichotomous exposures, there are a total of four exposure profiles (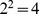) and a total of nine (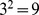) classes of sufficient causes [7], [8]. The classes of sufficient causes can be represented by a ternary string of length two, 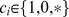 for 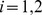, such that a class contains “
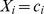
” as one of its component causes if 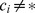, and does not involve 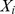 whatsoever if 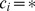, that is (see Figure 1), the all-unknown class (class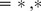), the “
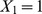
” class (class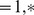), the “
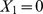
” class (class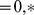), the “
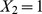
” class (class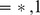), the “
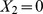
” class (class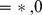), the “
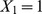
”
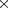
“
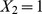
” interactive class (class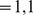), the “
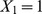
”
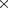
“
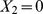
” interactive class (class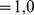), the “
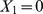
”
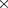
“
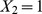
” interactive class (class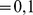), and the “
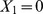
”
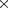
“
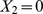
” interactive class (class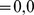) [8]. Let 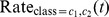 denote for the ‘class
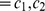
’ sufficient causes the (instantaneous) arrival rate for the unknown components (completion rate) at follow-up time 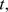 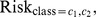 the cumulative completion risk (probability) in (0, 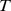), 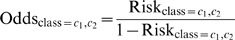 the cumulative completion odds in (0, 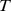), and 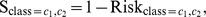 the probability of no completion in (0, 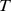).

As mentioned previously, a peril is simply a cumulative rate exponentiated. Without lost to follow up and competing death, a peril is also the inverse of a survival (risk complement) or one plus an odds (Exhibit S1), that is,
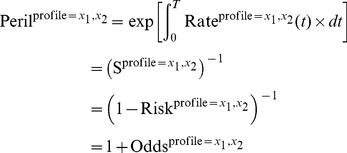
(1)for a ‘
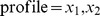
’ subject, and
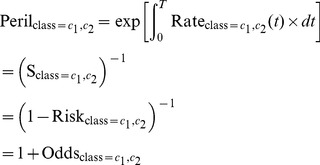
(2)for a ‘class
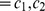
’ sufficient causes. A peril is dimensionless and ranges from 1 (no peril) to infinity (maximum peril).

I invoke the no redundancy assumption [8], [9] that in a sufficiently short time interval, (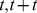), for each and every subject in the population there can only be at most one arrival event of the unknown components. Exhibit S2 shows that under such an assumption, a peril for a specific exposure profile is the product of four perils corresponding to the four ‘completable classes’ (defined in Exhibit S2) for that exposure profile, that is,
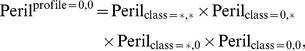
(3)

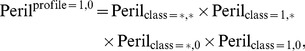
(4)

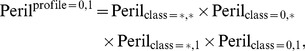
(5)and
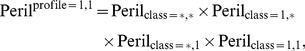
(6)respectively.

Next, define the peril ratio (PR) for a ‘
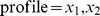
’ subject as
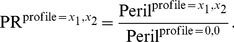
(7)


Because perils are the inverses of survivals, a peril ratio can be interpreted as the ‘fold decrease’ in survival (comparing ‘
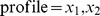
’ subject with ‘
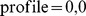
’ subject). Define PRISM as
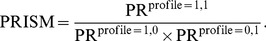
(8)


PRISM is a synergy index based on multiplicativity of peril ratios, or equivalently, multiplicativity of fold decreases in survivals. Intriguingly from Equations (3)∼(6), we see that the above defined PRISM can alternatively be expressed using the perils of the interactive classes only:
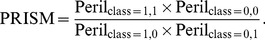
(9)


We will say there is synergism between 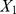 and 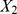 in sufficient cause sense, if at least one of 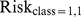, 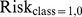, 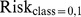, and 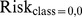 is non-zero (or equivalently, if at least one of 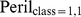, 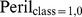, 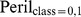, and 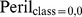 differs from one). Because there are a total 9 classes of sufficient causes but only a total of 4 exposure profiles, the class-specific perils (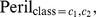
where 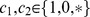) by themselves are not identifiable (not estimable from the data). However, this non-identifiability problem does not hamper our ability to test synergisms. A two-sided test on PRISM as defined in Equation (8),
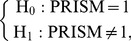
(10)is a global test for synergisms. (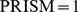 is the condition of no multiplicative interaction on the peril scale, or equivalently, no additive interaction on the cumulative rate scale.) The significance of the test implies the presence of at least one of the following four synergy classes: ‘class
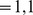
’, ‘class
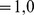
’, ‘class
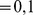
’ and ‘class
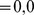
’. This is because from Equation (9), 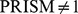 forbids 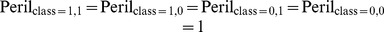 and therefore also 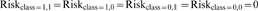 (Note however that 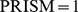 does not guarantee the absence of synergisms; a perfect cancellation of the positive and negative synergisms also leads to 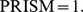)

A one-sided test on PRISM,
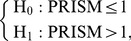
(11)is a test for positive synergisms of ‘class
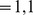
’ and ‘class
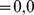
’ (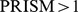 forbids 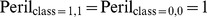 and 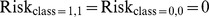), and a one-sided test,
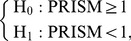
(12)a test for negative synergisms of ‘class
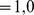
’ and ‘class
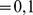
’ (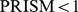 forbids 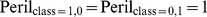 and 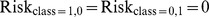). PRISM also permits a test specifically for a particular synergy class, albeit with a more stringent threshold. From Equations (3) and (9), we see that 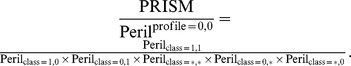 Therefore,
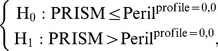
(13)is a test specifically for ‘class
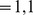
’ (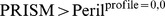 forbids 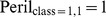 and 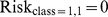). By similar arguments,
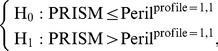
(14)is a test specifically for ‘class
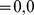
’,
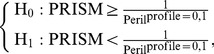
(15)a test specifically for ‘class
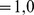
’, and
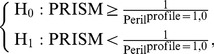
(16)a test specifically for ‘class
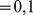
’, respectively.

**Figure 2:**
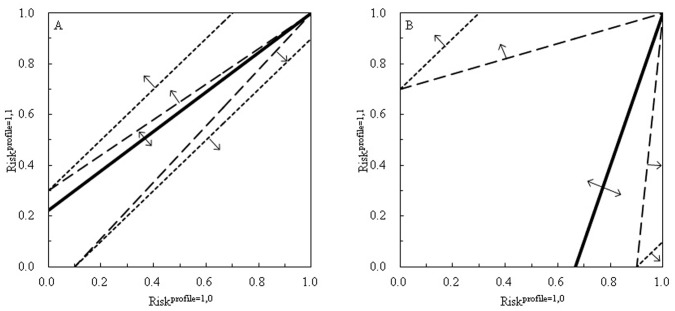
Thresholds for PRISM (peril ratio index of synergy based on multiplicativity) and RERI (relative excess risk due to interaction), when 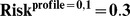 and 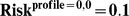 (A), and when 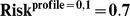 and 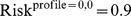 (B). The solid lines are the no-synergy lines of 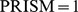, above which are the regions of positive synergisms, and below which, the regions of negative synergisms. The upper long-dash lines mark the thresholds of 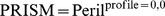, above which are the regions of the ‘class
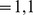
’ synergy. The lower long-dash lines mark the thresholds of 
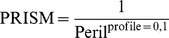
, below which are the regions of the ‘class
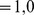
’ synergy. The upper short-dash lines mark the thresholds of 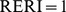, above which are the regions of the ‘class
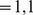
’ synergy. The lower short-dash lines mark the thresholds of 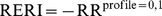, below which are the regions of the ‘class
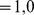
’ synergy.

Figure 2 presents the thresholds for the PRISM test. The solid lines are the no-synergy lines of 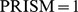. Above the lines are the regions of positive synergisms, and below it, the regions of negative synergisms. The upper long-dash lines mark the thresholds of 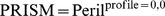, above which are the regions of the ‘class
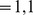
’ synergy, whereas the lower long-dash lines mark the thresholds of 
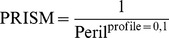
, below which are the regions of the ‘class
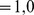
’ synergy.

A comparison of the above PRISM test with the RERI test [3]–[6] is in order. The former is based on cumulative rates, while the latter, cumulative risks. RERI can also test for specific synergisms: 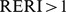 for ‘class
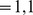
’ [corresponding to PRISM Test (13)], 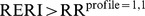 for ‘class
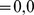
’ [corresponding to PRISM Test (14)], 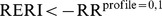 for ‘class
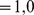
’ [corresponding to PRISM Test (15)], and 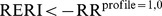 for ‘class
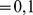
’ [corresponding to PRISM Test (16)], respectively.

In Figure 2, the upper short-dash lines mark the threshold of 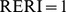 (for ‘class
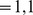
’), whereas the lower short-dash lines, the threshold of 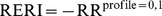 (for ‘class
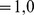
’). We see that the RERI thresholds are considerably more stringent than the corresponding PRISM thresholds (long-dashed lines). A proof is given in Exhibit S3 showing that a synergy can always be detected by PRISM if it is detected by RERI.

**Figure 3:**
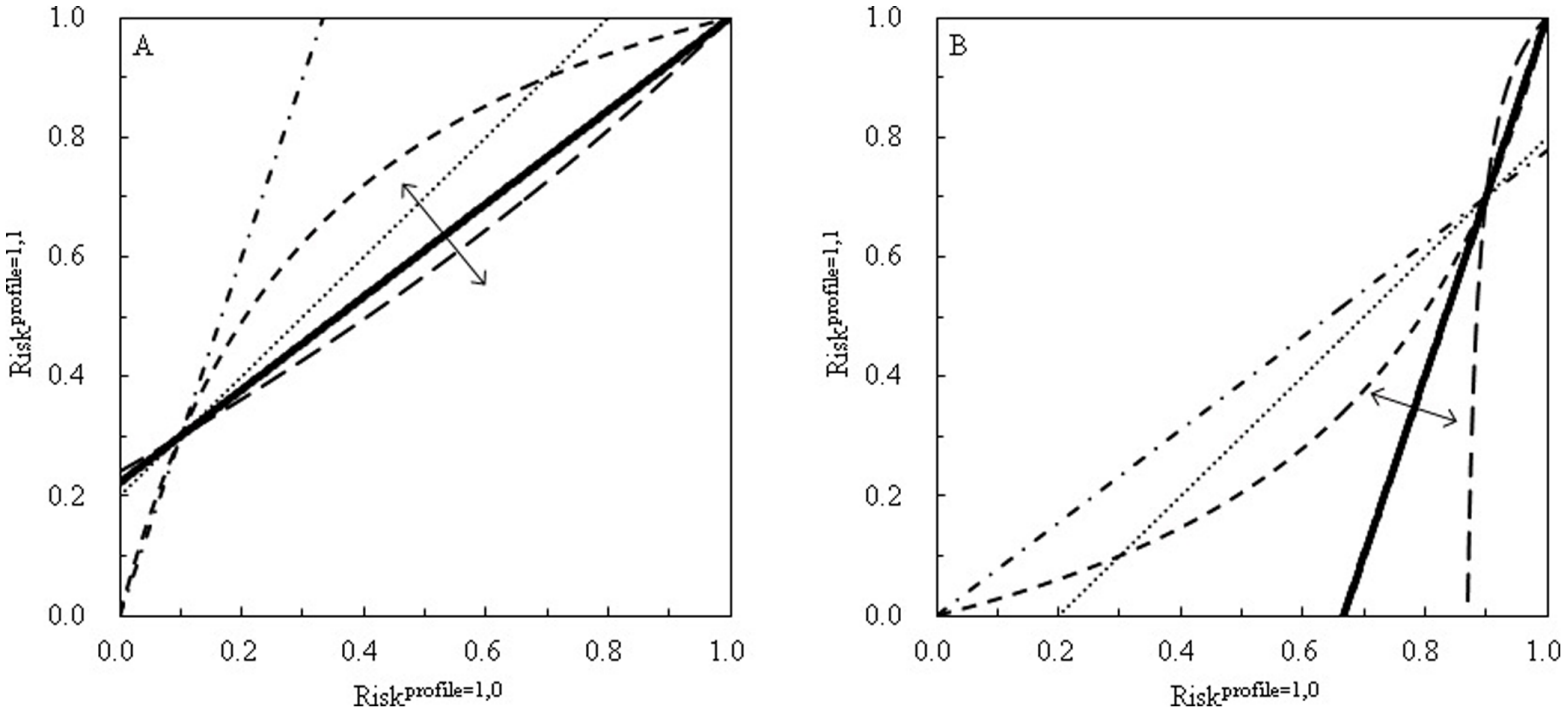
The no-synergy lines judged by the PRISM (peril ratio index of synergy based on multiplicativity), and the no-interaction lines judged by the SIM (synergistic index of multiplicativity) and RERI (relative excess risk due to interaction) criteria, when 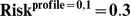 and 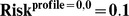 (A), and when 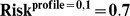 and 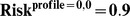 (B). The solid lines are the no-synergy lines of 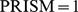, above which are the regions of positive synergisms, and below which, the regions of negative synergisms. The dash-and-dot lines are the no-interaction lines of 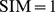 in terms of relative risks. The short-dash lines are the no-interaction lines of 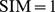 in terms of odds ratios. The dotted lines are the no-interaction lines of 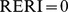 in terms of relative risks. The long-dash lines are the no-interaction lines of 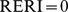 in terms of odds ratio.

It is also of interest to re-examine the situations in which there is not evidence of interaction judged, respectively, by the RERI index (in terms of risk ratios as the above, or odds ratios, 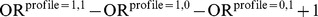), and the SIM index (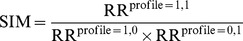 in terms of risk ratios, or 
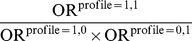
 in terms of odds ratios). From Figure 3, we see that a no-interaction line either of 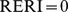 or 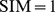 can penetrate deeply into the zones of positive (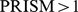, regions marked by the upward arrows) and negative (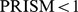, regions marked by the downward arrows) synergisms. This suggests that many situations in which there is not evidence of interaction previously judged by the traditional indices of SIM and RERI could in fact be *bona fide* positive or negative synergisms. Exhibit S4 shows that it is only when the disease under study is exceedingly rare or exceedingly common that a RERI criterion for rare diseases and a SIM criterion for common diseases shall correspond to the proposed PRISM criterion.

Sometimes, it is reasonable to assume the monotonicity assumption [10]–[12] that neither 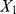 nor 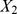 has a preventive action to disease, or more specifically, the assumption of no preventive action [13], [14] that component causes such as 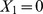 and 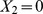 cannot be present in any class of sufficient causes (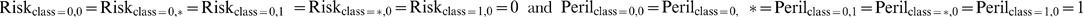). This leaves us now with only a total of 4 classes of sufficient causes, the perils of which are all identifiable. From Equations (3)∼(7), and (9), we see now that 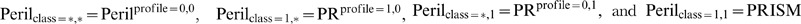. Therefore, the cumulative completion risks (probabilities) for the 4 classes of sufficient causes are:
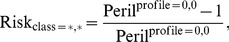
(17)

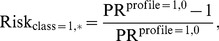
(18)

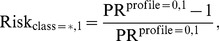
(19)and
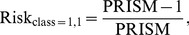
(20)respectively.

Exhibit S5 details all the necessary formulas (including the large-sample variances) for a synergy analysis in terms of cell counts of a study.

### An Example

As an example, I re-analyze a cohort study (the Example 3 in Zou’s paper [15]) using the PRISM approach. The cohort study assesses the effects of age (coded as 1 if age
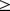
40 years and 0 if otherwise) and body mass index (BMI, coded as 1 if BMI
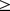
25 kg/m^2^ and 0 if otherwise) on hypertension (coded as 1 if diastolic blood pressure
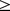
90 mmHg and 0 if otherwise). Table 1 presents the perils and the 95% confidence intervals (CIs) for the four exposure profiles. Using the (young age, low BMI) as the reference, the peril ratios and the 95% CIs are also presented, which show the fold decreases in hypertension-free probabilities. The PRISM for this example is calculated as 1.0905 with a 95% CI of 1.0341∼1.1499. This implies that the peril ratios in this example (fold decreases in hypertension-free probabilities) do not conform to a multiplicative model.

**Table 1:** The example data (Reference 15), the perils, the peril ratios and the peril ratio index of synergy based on multiplicativity (PRISM) with the 95% confidence intervals (CIs).

Age^a^	BMI^b^	Hypertension		Peril (95% CI)	Peril Ratio(95% CI)
		No	Yes		
young	low	1731	79	1.0456 (1.0364∼1.0572)	1.0000
old	low	581	100	1.1721 (1.1392∼1.2128)	1.1210 (1.0849∼1.1582)
young	high	1232	153	1.1242 (1.1050∼1.1469)	1.0751 (1.0528∼1.0979)
old	high	743	278	1.3742 (1.3260∼1.4294)	1.3142 (1.2642∼1.3662)
PRISM (95% CI) = 1.0905 (1.0341∼1.1499)					

a old: age
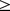
40 years; young: age
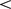
40.

b BMI: body mass index; high: BMI
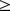
25 kg/m^2^; low: BMI
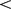
25.

The test statistic of the global test for synergisms for this example is 3.1961 with a highly significant two-sided p-value of 0.0014 (for global synergisms) and a highly significant one-sided (upper tail) p-value of 0.0007 (for positive synergisms). The specific test for ‘class
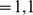
’ (the synergy between old age and high BMI in this example) has a test statistic of 1.5763 with a marginally insignificant p-value of 0.0575. (The test for the same synergy class using the RERI approach yields a comparatively much larger p-value of 0.2399.) Note that this example is used for illustrative purposes only and should not be taken as evidence of actual synergism here since the exposures have been dichotomized. Assessing synergism or interaction for continuous exposures under dichotomization is considerably trickier [16].

If the assumption of no preventive action is deemed reasonable for this example, the cumulative completion risks and the 95% CIs can be calculated for the four classes of sufficient causes as presented in Table 2.

**Table 2:** The cumulative completion risks with the 95% confidence intervals (CIs) for the example data in Table 1.

Class of Sufficient Causes	Cumulative Completion Risk(95% CI)
All Unknown	4.36% (3.51%∼5.41%)
Old Age^a^	10.79% (8.20%∼14.07%)
High BMI^b^	6.99% (5.27%∼9.21%)
“Old Age” 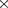 “High BMI^b^”	8.30% (4.55%∼14.64%)

a age
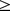
40 years.

b body mass index
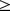
25 kg/m^2^.

## Discussion

To study toxic effects of two chemicals administered simultaneously, the model of simple independent action had seen a very long history of use in toxicopharmacology dating back to 1939 [17]. In recent decades, epidemiologists [11], [12], [18]–[22] and researchers in other fields (infectious disease [23], genetics [24] and environmental health [25]) also began to define interactions based on deviation from independence. However, independence is a rather strong assumption. In the present context of sufficient component causes, the completions of different classes of sufficient causes are not likely to be independent events. Rather, they are more likely to be positively correlated to one another due to possible overlapping of the constituent factors of the class-specific unknowns. This paper replaces the independence assumption with a much weaker Poisson-like assumption–the no redundancy assumption. The assumption dictates that in a sufficiently short time interval, for each and every subject in the population the probability of two or more than two arrival events of the unknown components is negligible. (Suzuki et al [14] previously introduced the concept of potential completion times of sufficient causes and assumed that each potential completion time is different. This is a different way to invoke the same no redundancy assumption.) Even with strong dependency in the arrival events, the no redundancy assumption should still hold in each and every time interval that is infinitesimally small, unless one argues that the probability is non-negligible that an overlapping constituent factor happens to be the last one to arrive, and in not just one but at least two classes of sufficient causes.

The assumption of proportional hazards (rates) has often been invoked in longitudinal follow-up studies (cohort studies) [8], [11], [12], [26], [27]. The assumption is often true (or approximately so) for most situations. But occasionally, we will see a larger deviation. For example, the hazard curves for different exposure profiles [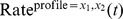 for 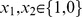] can cross each other, thus failing the assumption completely. The proposed PRISM criterion does not need the proportional hazards assumption. It is a valid synergy test irrespectively of proportionalities, non-proportionalities or crossings of the hazard curves. If the hazard curve for each and every exposure profile in population A is a constant multiple (say, 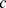) of the corresponding hazard curve in population B, we will have 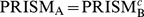 (Exhibit S6) and therefore achieve the same conclusion about synergisms (apart from statistical variations) in the two populations.

In a recent paper, VanderWeele [27] studied proportional hazards models and made an interesting conclusion that “causal interactions can disappear as time progresses, ie, whether a causal interaction is present depends on the follow-up time”. Exhibit S7 re-examines this problem using the proposed PRISM criterion. It is found that in the proportional hazards models, theoretically a synergy signal will not go away with more follow-up times. However, there does exist an optimal follow-up time for maximum power of the PRISM test: to follow up the cohort subjects until ∼80% of them are diseased (assuming no lost to follow up and competing death).

In this paper, methods for assessing sufficient cause synergism for rates without the co-cause independence and monotonicity assumptions are presented. The method can be extended in several ways. First, it is worthwhile to extend the present method to deal with exposures with multiple levels. This will allow us to study dose-response exposure-disease relations as well as dose-dependent causal mechanistic interactions. Second, casting the present method in a proper modeling framework should also prove useful to accommodate more than two exposures and to adjust for possible confounders. Here we need to go beyond the commonly used logistic regression (in epidemiology [7]) and probit regression (in econometrics [28]) for binary outcomes, because these two models are purely statistical in nature without a built-in causal mechanism. Third, asides from confounding, selection bias and measurement errors, a cohort study can be complicated by the problems of lost to follow up and competing deaths, etc. A valid synergy analysis for censored data also awaits further studies. Finally, in an ordinary case-control study for a rare disease, the PRISM criterion can be approximated by the RERI criterion in terms of odds ratios (Exhibit S4). Without the rare-disease assumption however, one may need to resort to alternative control sampling schemes, such as density sampling or case-base (case-cohort) sampling [7]. How to test causal mechanistic interactions under such settings also deserves further studies.

## Supporting Information

Exhibit S1 **Relation between exponentiated cumulative rate and other indices.**
(DOC)

Exhibit S2 **Relation between profile-specific perils/rates and class-specific perils/rates.**
(DOC)

Exhibit S3 **A synergy can always be detected by PRISM if it is detected by RERI.**
(DOC)

Exhibit S4 **PRISM criterion under extreme conditions.**
(DOC)

Exhibit S5 **Formulas for synergy analysis in terms of cell counts.**
(DOC)

Exhibit S6 **PRISM indices for two populations with proportional hazards.**
(DOC)

Exhibit S7 **Synergy signal and follow-up time.**
(DOC)
