# Supplementary material for: Assessing Causal Mechanistic Interactions: A Peril Ratio Index of Synergy Based on Multiplicativity
Source: PLoS One. 2013 Jun 24;8(6):e67424. doi: 10.1371/journal.pone.0067424 (PMC3691192; doi:10.1371/journal.pone.0067424)
Supplement: Exhibit S1 — Relation between exponentiated cumulative rate and other indices. (DOC) [file pone.0067424.s001.doc]

Supporting Information of

Assessing Causal Mechanistic Interactions: a Peril Ratio Index of Synergy based on Multiplicativity

Author: Wen-Chung Lee^1,2^

Author’s affiliation: 1. Research Center for Genes, Environment and Human Health,

College of Public Health, National Taiwan University, Taipei, Taiwan.

2. Institute of Epidemiology and Preventive Medicine,

College of Public Health, National Taiwan University, Taipei, Taiwan.

Correspondence & reprint requests: Prof. Wen-Chung Lee,

Rm. 536, No. 17, Xuzhou Rd., Taipei 100, Taiwan.

(FAX: 886-2-23511955)

(e-mail:wenchung@ntu.edu.tw)

Exhibit S1. Relation between exponentiated cumulative rate and other indices.

By definition, an instantaneous rate is $\text{Rate}(t)=\frac{\text{ number of events }}{\text{total person-time}}=\frac{-N\times d\text{S}(t)}{N\times\text{S}(t)\times dt}$, where $N$ is the total number of subjects. Thus we have,

$\text{Rate}(t)\times dt=-\frac{1}{\text{S}(t)}\times d\text{S}(t).$ (S1.1)

Integrating from 0 to $T$ on both sides of (S1.1), we achieve

$\begin{matrix} \int_{0}^{T} \text{Rate}(t)\times dt=-\int_{0}^{T} \frac{1}{\text{S}(t)}\times d\text{S}(t) \\ \text{ }=-\log\text{S}(T). \end{matrix}$ (S1.2)

Exponentiating both sides of (S1.2), we arrive at

$\exp\left[\int_{0}^{T} \text{Rate}(t)\times dt\right]=\frac{1}{\text{S}(T)}.$ (S1.3)

From (S1.3), simple algebra leads to Equations (1) and (2) in text.
