# Supplementary material for: Assessing Causal Mechanistic Interactions: A Peril Ratio Index of Synergy Based on Multiplicativity
Source: PLoS One. 2013 Jun 24;8(6):e67424. doi: 10.1371/journal.pone.0067424 (PMC3691192; doi:10.1371/journal.pone.0067424)
Supplement: Exhibit S2 — Relation between profile-specific perils/rates and class-specific perils/rates. (DOC) [file pone.0067424.s002.doc]

Supporting Information of

Assessing Causal Mechanistic Interactions: a Peril Ratio Index of Synergy based on Multiplicativity

Author: Wen-Chung Lee^1,2^

Author’s affiliation: 1. Research Center for Genes, Environment and Human Health,

College of Public Health, National Taiwan University, Taipei, Taiwan.

2. Institute of Epidemiology and Preventive Medicine,

College of Public Health, National Taiwan University, Taipei, Taiwan.

Correspondence & reprint requests: Prof. Wen-Chung Lee,

Rm. 536, No. 17, Xuzhou Rd., Taipei 100, Taiwan.

(FAX: 886-2-23511955)

(e-mail:wenchung@ntu.edu.tw)

Exhibit S2. Relation between profile-specific perils/rates and class-specific perils/rates.

Lee [8] previously defined the completion sufficient cause types. A subject with the unknown in the ‘class$=c_{1},c_{2}$’ arriving in ($t,\text{ }t+\triangle t$) is of the ‘ready$=c_{1},c_{2}$’ type at time $t$. If none of the nine possible arrival events occurs in ($t,\text{ }t+\triangle t$) for a subject, he/she is of the ‘ready$=$none’ type at time $t$. Let $p_{\text{ready}=c_{1},c_{2}}(t)$ for $c_{1},c_{2}\in\left\{1,0,*\right\}$ denote the proportion of subjects of the ‘ready$=c_{1},c_{2}$’ type at time $t$ in the population. [The proportion of subjects of the ‘ready$=$none’ type at time $t$ in the population is $p_{\text{ready}=\text{none}}(t)=1-\sum_{c_{1},c_{2}\in\left\{1,0,*\right\}} p_{\text{ready}=c_{1},c_{2}}(t).$]

For people exposed to neither factor ($X_{1}=X_{2}=0$), they can develop the disease in ($t,\text{ }t+\triangle t$) because of the completion of the ‘class$=*,*$’, ‘class$=0,*$’, ‘class$=*,0$’ and ‘class$=0,0$’ sufficient causes. (These four classes are the completable classes for them.) Under the no redundancy assumption, the disease probability for these people in ($t,\text{ }t+\triangle t$) where $\triangle t\to0$ is therefore $\text{Rate}^{\text{profile}=0,0}(t)\times\triangle t$ $=p_{\text{ready}=*,*}(t)+p_{\text{ready}=0,*}(t)+p_{\text{ready}=*,0}(t)+p_{\text{ready}=0,0}(t)$ $=\text{Rate}_{\text{class}=*,*}(t)\times\triangle t+\text{Rate}_{\text{class}=0,*}(t)\times\triangle t+\text{Rate}_{\text{class}=*,0}(t)\times\triangle t+\text{Rate}_{\text{class}=0,0}(t)\times\triangle t.$ Thus we have

$\text{Rate}^{\text{profile}=0,0}(t)=\text{Rate}_{\text{class}=*,*}(t)+\text{Rate}_{\text{class}=0,*}(t)+\text{Rate}_{\text{class}=*,0}(t)+\text{Rate}_{\text{class}=0,0}(t).$ (S2.1)

In a similar vein, we have

$\text{Rate}^{\text{profile}=1,0}(t)=\text{Rate}_{\text{class}=*,*}(t)+\text{Rate}_{\text{class}=1,*}(t)+\text{Rate}_{\text{class}=*,0}(t)+\text{Rate}_{\text{class}=1,0}(t),$ (S2.2)

$\text{Rate}^{\text{profile}=0,1}(t)=\text{Rate}_{\text{class}=*,*}(t)+\text{Rate}_{\text{class}=0,*}(t)+\text{Rate}_{\text{class}=*,1}(t)+\text{Rate}_{\text{class}=0,1}(t),$ (S2.3)

and

$\text{Rate}^{\text{profile}=1,1}(t)=\text{Rate}_{\text{class}=*,*}(t)+\text{Rate}_{\text{class}=1,*}(t)+\text{Rate}_{\text{class}=*,1}(t)+\text{Rate}_{\text{class}=1,1}(t).$ (S2.4)

Noting that a peril is an exponentiated cumulative rate [Equations (1) and (2) in text], we integrate (from 0 to $T$) and then exponentiate both sides of Equations (S2.1)~(S2.4) to yield Equations (3)~(6) in text, respectively.
