# Supplementary material for: Assessing Causal Mechanistic Interactions: A Peril Ratio Index of Synergy Based on Multiplicativity
Source: PLoS One. 2013 Jun 24;8(6):e67424. doi: 10.1371/journal.pone.0067424 (PMC3691192; doi:10.1371/journal.pone.0067424)
Supplement: Exhibit S3 — A synergy can always be detected by PRISM if it is detected by RERI. (DOC) [file pone.0067424.s003.doc]

Supporting Information of

Assessing Causal Mechanistic Interactions: a Peril Ratio Index of Synergy based on Multiplicativity

Author: Wen-Chung Lee^1,2^

Author’s affiliation: 1. Research Center for Genes, Environment and Human Health,

College of Public Health, National Taiwan University, Taipei, Taiwan.

2. Institute of Epidemiology and Preventive Medicine,

College of Public Health, National Taiwan University, Taipei, Taiwan.

Correspondence & reprint requests: Prof. Wen-Chung Lee,

Rm. 536, No. 17, Xuzhou Rd., Taipei 100, Taiwan.

(FAX: 886-2-23511955)

(e-mail:wenchung@ntu.edu.tw)

Exhibit S3. A synergy can always be detected by PRISM if it is detected by RERI.

To detect ‘class$=1,1$’, the criterion $\text{PRISM}>\text{Peril}^{\text{profile}=0,0}$ [Test (13) in text] amounts to [see Equations (1) and (8) in text]

$\left(1-\text{Risk}^{\text{profile}=1,1}\right)^{-1}>\left(1-\text{Risk}^{\text{profile}=1,0}\right)^{-1}\times\left(1-\text{Risk}^{\text{profile}=0,1}\right)^{-1}$, (S3.1)

and the criterion $\text{RERI}>1$ (References 3-6) amounts to

$\text{Risk}^{\text{profile}=1,1}>\text{Risk}^{\text{profile}=1,0}+\text{Risk}^{\text{profile}=0,1}$. (S3.2)

Upon rearrangement of the terms, (S3.1) becomes

$\text{Risk}^{\text{profile}=1,1}+\text{Risk}^{\text{profile}=1,0}\times\text{Risk}^{\text{profile}=0,1}>\text{Risk}^{\text{profile}=1,0}+\text{Risk}^{\text{profile}=0,1}.$ (S3.3)

Because (S3.2) implies (S3.3), we see that $\text{PRISM}>\text{Peril}^{\text{profile}=0,0}$ must be true if $\text{RERI}>1$ is true. Similar arguments apply to other classes.
