# Supplementary material for: Assessing Causal Mechanistic Interactions: A Peril Ratio Index of Synergy Based on Multiplicativity
Source: PLoS One. 2013 Jun 24;8(6):e67424. doi: 10.1371/journal.pone.0067424 (PMC3691192; doi:10.1371/journal.pone.0067424)
Supplement: Exhibit S4 — PRISM criterion under extreme conditions. (DOC) [file pone.0067424.s004.doc]

Supporting Information of

Assessing Causal Mechanistic Interactions: a Peril Ratio Index of Synergy based on Multiplicativity

Author: Wen-Chung Lee^1,2^

Author’s affiliation: 1. Research Center for Genes, Environment and Human Health,

College of Public Health, National Taiwan University, Taipei, Taiwan.

2. Institute of Epidemiology and Preventive Medicine,

College of Public Health, National Taiwan University, Taipei, Taiwan.

Correspondence & reprint requests: Prof. Wen-Chung Lee,

Rm. 536, No. 17, Xuzhou Rd., Taipei 100, Taiwan.

(FAX: 886-2-23511955)

(e-mail:wenchung@ntu.edu.tw)

Exhibit S4. PRISM criterion under extreme conditions.

Because a peril is one plus an odds [see Equation (1) in text], the criterion of $\text{PRISM}=1$ [see Equation (8) and Test (10) in text] is

$\frac{\left(1+\text{Odds}^{\text{profile}=1,1}\right)\times\left(1+\text{Odds}^{\text{profile}=0,0}\right)}{\left(1+\text{Odds}^{\text{profile}=1,0}\right)\times\left(1+\text{Odds}^{\text{profile}=0,1}\right)}=1.$ (S4.1)

Taking natural logarithm to both sides of (S4.1), we obtain $\log\left(1+\text{Odds}^{\text{profile}=1,1}\right)-\log\left(1+\text{Odds}^{\text{profile}=1,0}\right)-\log\left(1+\text{Odds}^{\text{profile}=0,1}\right)+\log\left(1+\text{Odds}^{\text{profile}=0,0}\right)=0.$ This becomes $\text{Odds}^{\text{profile}=1,1}-\text{Odds}^{\text{profile}=1,0}-\text{Odds}^{\text{profile}=0,1}+\text{Odds}^{\text{profile}=0,0}=0$ for rare diseases [$\log\left(1+x\right)\approx x$ when $x\to0$], and upon being further divided by $\text{Odds}^{\text{profile}=0,0}$ on both sides, $\text{OR}^{\text{profile}=1,1}-\text{OR}^{\text{profile}=1,0}-\text{OR}^{\text{profile}=0,1}+1=0,$ where ORs are the odds ratios with ‘$\text{profile}=0,0$’ as the reference. This is approximately the criterion of $\text{RERI}=0$ (References 3-6), since odds ratios and risk ratios are approximately equal for rare diseases.

At the other extreme when the disease under study is exceedingly common (with disease risks tending to one, or disease odds, to infinity), (S4.1) becomes $\frac{\text{Odds}^{\text{profile}=1,1}\times\text{Odds}^{\text{profile}=0,0}}{\text{Odds}^{\text{profile}=1,0}\times\text{Odds}^{\text{profile}=0,1}}=1$ or $\frac{\text{OR}^{\text{profile}=1,1}}{\text{OR}^{\text{profile}=1,0}\times\text{OR}^{\text{profile}=0,1}}=1.$
