# Supplementary material for: Assessing Causal Mechanistic Interactions: A Peril Ratio Index of Synergy Based on Multiplicativity
Source: PLoS One. 2013 Jun 24;8(6):e67424. doi: 10.1371/journal.pone.0067424 (PMC3691192; doi:10.1371/journal.pone.0067424)
Supplement: Exhibit S5 — Formulas for synergy analysis in terms of cell counts. (DOC) [file pone.0067424.s005.doc]

Supporting Information of

Assessing Causal Mechanistic Interactions: a Peril Ratio Index of Synergy based on Multiplicativity

Author: Wen-Chung Lee^1,2^

Author’s affiliation: 1. Research Center for Genes, Environment and Human Health,

College of Public Health, National Taiwan University, Taipei, Taiwan.

2. Institute of Epidemiology and Preventive Medicine,

College of Public Health, National Taiwan University, Taipei, Taiwan.

Correspondence & reprint requests: Prof. Wen-Chung Lee,

Rm. 536, No. 17, Xuzhou Rd., Taipei 100, Taiwan.

(FAX: 886-2-23511955)

(e-mail:wenchung@ntu.edu.tw)

Exhibit S5. Formulas for synergy analysis in terms of cell counts.

The cell counts:

____________________________________________________

$X_{1}$ $X_{2}$ $D=0$ $D=1$ Total

____________________________________________________

0 0 $a$ $b$ $N_{00}=a+b$

1 0 $c$ $d$ $N_{10}=c+d$

0 1 $e$ $f$ $N_{01}=e+f$

1 1 $g$ $h$ $N_{11}=g+h$

____________________________________________________

1. Point Estimates:

Point estimates for the disease odds are

$$\widehat{\text{Odds}}^{\text{profile}=0,0}=\frac{b}{a},$$

$$\widehat{\text{Odds}}^{\text{profile}=1,0}=\frac{d}{c},$$

$$\widehat{\text{Odds}}^{\text{profile}=0,1}=\frac{f}{e},$$

$$\widehat{\text{Odds}}^{\text{profile}=1,1}=\frac{h}{g}.$$

Point estimates for the perils are

$$\widehat{\text{Peril}}^{\text{profile}=0,0}=1+\widehat{\text{Odds}}^{\text{profile}=0,0},$$

$$\widehat{\text{Peril}}^{\text{profile}=1,0}=1+\widehat{\text{Odds}}^{\text{profile}=1,0},$$

$$\widehat{\text{Peril}}^{\text{profile}=0,1}=1+\widehat{\text{Odds}}^{\text{profile}=0,1},$$

$$\widehat{\text{Peril}}^{\text{profile}=1,1}=1+\widehat{\text{Odds}}^{\text{profile}=1,1}.$$

Point estimates for the peril ratios are

$$\widehat{\text{PR}}^{\text{profile}=1,1}=\frac{\widehat{\text{Peril}}^{\text{profile}=1,1}}{\widehat{\text{Peril}}^{\text{profile}=0,0}},$$

$$\widehat{\text{PR}}^{\text{profile}=1,0}=\frac{\widehat{\text{Peril}}^{\text{profile}=1,0}}{\widehat{\text{Peril}}^{\text{profile}=0,0}},$$

$$\widehat{\text{PR}}^{\text{profile}=0,1}=\frac{\widehat{\text{Peril}}^{\text{profile}=0,1}}{\widehat{\text{Peril}}^{\text{profile}=0,0}}.$$

Point estimate for the PRISM is

$$\widehat{\text{PRISM}}=\frac{\widehat{\text{PR}}^{\text{profile}=1,1}}{\widehat{\text{PR}}^{\text{profile}=1,0}\times\widehat{\text{PR}}^{\text{profile}=0,1}}=\frac{\widehat{\text{Peril}}^{\text{profile}=1,1}\times\widehat{\text{Peril}}^{\text{profile}=0,0}}{\widehat{\text{Peril}}^{\text{profile}=1,0}\times\widehat{\text{Peril}}^{\text{profile}=0,1}}.$$

Under the assumption of no preventive action, point estimates for the cumulative completion risks are

$$\widehat{\text{Risk}}_{\text{class}=*,*}=\frac{\widehat{\text{Peril}}^{\text{profile}=0,0}-1}{\widehat{\text{Peril}}^{\text{profile}=0,0}},$$

$$\widehat{\text{Risk}}_{\text{class}=1,*}=\frac{\widehat{\text{PR}}^{\text{profile}=1,0}-1}{\widehat{\text{PR}}^{\text{profile}=1,0}},$$

$$\widehat{\text{Risk}}_{\text{class}=*,1}=\frac{\widehat{\text{PR}}^{\text{profile}=0,1}-1}{\widehat{\text{PR}}^{\text{profile}=0,1}},$$

$$\widehat{\text{Risk}}_{\text{class}=1,1}=\frac{\widehat{\text{PRISM}}-1}{\widehat{\text{PRISM}}}.$$

1. Variances:

Variances of the log disease odds are

$$\text{Var}\left(\log\widehat{\text{Odds}}^{\text{profile}=0,0}\right)=\frac{1}{a}+\frac{1}{b},$$

$$\text{Var}\left(\log\widehat{\text{Odds}}^{\text{profile}=1,0}\right)=\frac{1}{c}+\frac{1}{d},$$

$$\text{Var}\left(\log\widehat{\text{Odds}}^{\text{profile}=0,1}\right)=\frac{1}{e}+\frac{1}{f},$$

$$\text{Var}\left(\log\widehat{\text{Odds}}^{\text{profile}=1,1}\right)=\frac{1}{g}+\frac{1}{h}.$$

Variance of the log perils are

$$\text{Var}\left(\log\widehat{\text{Peril}}^{\text{profile}=0,0}\right)=\frac{\widehat{\text{Odds}}^{\text{profile}=0,0}}{N_{00}},$$

$$\text{Var}\left(\log\widehat{\text{Peril}}^{\text{profile}=1,0}\right)=\frac{\widehat{\text{Odds}}^{\text{profile}=1,0}}{N_{10}},$$

$$\text{Var}\left(\log\widehat{\text{Peril}}^{\text{profile}=0,1}\right)=\frac{\widehat{\text{Odds}}^{\text{profile}=0,1}}{N_{01}},$$

$$\text{Var}\left(\log\widehat{\text{Peril}}^{\text{profile}=1,1}\right)=\frac{\widehat{\text{Odds}}^{\text{profile}=1,1}}{N_{11}}.$$

Variances of the log peril ratios are

$$\text{Var}\left(\log\widehat{\text{PR}}^{\text{profile}=1,1}\right)=\text{Var}\left(\log\widehat{\text{Peril}}^{\text{profile}=1,1}\right)+\text{Var}\left(\log\widehat{\text{Peril}}^{\text{profile}=0,0}\right),$$

$$\text{Var}\left(\log\widehat{\text{PR}}^{\text{profile}=1,0}\right)=\text{Var}\left(\log\widehat{\text{Peril}}^{\text{profile}=1,0}\right)+\text{Var}\left(\log\widehat{\text{Peril}}^{\text{profile}=0,0}\right),$$

$$\text{Var}\left(\log\widehat{\text{PR}}^{\text{profile}=0,1}\right)=\text{Var}\left(\log\widehat{\text{Peril}}^{\text{profile}=0,1}\right)+\text{Var}\left(\log\widehat{\text{Peril}}^{\text{profile}=0,0}\right).$$

Variance of the log PRISM is

$$\begin{matrix} \text{Var}\left(\log\widehat{\text{PRISM}}\right)=\text{Var}\left(\log\widehat{\text{Peril}}^{\text{profile}=1,1}\right)+\text{Var}\left(\log\widehat{\text{Peril}}^{\text{profile}=0,0}\right) \\ \text{ }+\text{Var}\left(\log\widehat{\text{Peril}}^{\text{profile}=1,0}\right)+\text{Var}\left(\log\widehat{\text{Peril}}^{\text{profile}=0,1}\right). \end{matrix}$$

1. Test Statistics:

Tests (10)~(12) in text are equivalent to testing for $\log\text{PRISM}\neq0$ (Test 10), $\log\text{PRISM}>0$ (Test 11) and $\log\text{PRISM}<0$ (Test 12), respectively, with a test statistic $Z_{\text{global}}=\frac{\log\widehat{\text{PRISM}}}{\sqrt{\text{Var}\left(\log\widehat{\text{PRISM}}\right)}}$ distributed as a standard normal distribution.

Test (13) in text is equivalent to testing for $\log\left(\frac{\text{Peril}^{\text{profile}=1,1}}{\text{Peril}^{\text{profile}=1,0}\times\text{Peril}^{\text{profile}=0,1}}\right)>0$, with a test statistic $Z_{\text{class}=1,1}=\frac{\log\widehat{\text{Peril}}^{\text{profile}=1,1}-\log\widehat{\text{Peril}}^{\text{profile}=1,0}-\log\widehat{\text{Peril}}^{\text{profile}=0,1}}{\sqrt{\text{Var}\left(\log\widehat{\text{Peril}}^{\text{profile}=1,1}\right)+\text{Var}\left(\log\widehat{\text{Peril}}^{\text{profile}=1,0}\right)+\text{Var}\left(\log\widehat{\text{Peril}}^{\text{profile}=0,1}\right)}}$ distributed as a standard normal distribution.

Test (14) in text is equivalent to testing for $\log\left(\frac{\text{Peril}^{\text{profile}=0,0}}{\text{Peril}^{\text{profile}=1,0}\times\text{Peril}^{\text{profile}=0,1}}\right)>0$, with a test statistic $Z_{\text{class}=0,0}=\frac{\log\widehat{\text{Peril}}^{\text{profile}=0,0}-\log\widehat{\text{Peril}}^{\text{profile}=1,0}-\log\widehat{\text{Peril}}^{\text{profile}=0,1}}{\sqrt{\text{Var}\left(\log\widehat{\text{Peril}}^{\text{profile}=0,0}\right)+\text{Var}\left(\log\widehat{\text{Peril}}^{\text{profile}=1,0}\right)+\text{Var}\left(\log\widehat{\text{Peril}}^{\text{profile}=0,1}\right)}}$ distributed as a standard normal distribution.

Test (15) in text is equivalent to testing for $\log\left(\frac{\text{Peril}^{\text{profile}=1,1}\times\text{Peril}^{\text{profile}=0,0}}{\text{Peril}^{\text{profile}=1,0}}\right)<0$, with a test statistic $Z_{\text{class}=1,0}=\frac{\log\widehat{\text{Peril}}^{\text{profile}=1,1}+\log\widehat{\text{Peril}}^{\text{profile}=0,0}-\log\widehat{\text{Peril}}^{\text{profile}=1,0}}{\sqrt{\text{Var}\left(\log\widehat{\text{Peril}}^{\text{profile}=1,1}\right)+\text{Var}\left(\log\widehat{\text{Peril}}^{\text{profile}=0,0}\right)+\text{Var}\left(\log\widehat{\text{Peril}}^{\text{profile}=1,0}\right)}}$ distributed as a standard normal distribution.

Test (16) in text is equivalent to testing for $\log\left(\frac{\text{Peril}^{\text{profile}=1,1}\times\text{Peril}^{\text{profile}=0,0}}{\text{Peril}^{\text{profile}=0,1}}\right)<0$, with a test statistic $Z_{\text{class}=0,1}=\frac{\log\widehat{\text{Peril}}^{\text{profile}=1,1}+\log\widehat{\text{Peril}}^{\text{profile}=0,0}-\log\widehat{\text{Peril}}^{\text{profile}=0,1}}{\sqrt{\text{Var}\left(\log\widehat{\text{Peril}}^{\text{profile}=1,1}\right)+\text{Var}\left(\log\widehat{\text{Peril}}^{\text{profile}=0,0}\right)+\text{Var}\left(\log\widehat{\text{Peril}}^{\text{profile}=0,1}\right)}}$ distributed as a standard normal distribution.

1. Confidence Intervals:

The 95% confidences intervals for the perils are

$$1+\exp\left\{\log\left(\widehat{\text{Peril}}^{\text{profile}=0,0}-1\right)\pm1.96\times\sqrt{\text{Var}\left(\log\widehat{\text{Odds}}^{\text{profile}=0,0}\right)}\right\},$$

$$1+\exp\left\{\log\left(\widehat{\text{Peril}}^{\text{profile}=1,0}-1\right)\pm1.96\times\sqrt{\text{Var}\left(\log\widehat{\text{Odds}}^{\text{profile}=1,0}\right)}\right\},$$

$$1+\exp\left\{\log\left(\widehat{\text{Peril}}^{\text{profile}=0,1}-1\right)\pm1.96\times\sqrt{\text{Var}\left(\log\widehat{\text{Odds}}^{\text{profile}=0,1}\right)}\right\},$$

$$1+\exp\left\{\log\left(\widehat{\text{Peril}}^{\text{profile}=1,1}-1\right)\pm1.96\times\sqrt{\text{Var}\left(\log\widehat{\text{Odds}}^{\text{profile}=1,1}\right)}\right\}.$$

The 95% confidences intervals for the peril ratios are

$$\exp\left[\log\widehat{\text{PR}}^{\text{profile}=1,1}\pm1.96\times\sqrt{\text{Var}\left(\log\widehat{\text{PR}}^{\text{profile}=1,1}\right)}\right],$$

$$\exp\left[\log\widehat{\text{PR}}^{\text{profile}=1,0}\pm1.96\times\sqrt{\text{Var}\left(\log\widehat{\text{PR}}^{\text{profile}=1,0}\right)}\right],$$

$$\exp\left[\log\widehat{\text{PR}}^{\text{profile}=0,1}\pm1.96\times\sqrt{\text{Var}\left(\log\widehat{\text{PR}}^{\text{profile}=0,1}\right)}\right].$$

The 95% confidences interval for the PRISM is

$$\exp\left[\log\widehat{\text{PRISM}}\pm1.96\times\sqrt{\text{Var}\left(\log\widehat{\text{PRISM}}\right)}\right].$$

Under the assumption of no preventive action, the 95% confidences intervals for the cumulative completion risks are (with both the numerator and the denominator taking the same sign):

$$\frac{\exp\left[\log\left(\frac{\widehat{\text{Risk}}_{\text{class}=*,*}}{1-\widehat{\text{Risk}}_{\text{class}=*,*}}\right)\pm\frac{1.96}{\widehat{\text{Risk}}_{\text{class}=*,*}}\times\sqrt{\text{Var}\left(\log\widehat{\text{Peril}}^{\text{profile}=0,0}\right)}\right]}{1+\exp\left[\log\left(\frac{\widehat{\text{Risk}}_{\text{class}=*,*}}{1-\widehat{\text{Risk}}_{\text{class}=*,*}}\right)\pm\frac{1.96}{\widehat{\text{Risk}}_{\text{class}=*,*}}\times\sqrt{\text{Var}\left(\log\widehat{\text{Peril}}^{\text{profile}=0,0}\right)}\right]},$$

$$\frac{\exp\left[\log\left(\frac{\widehat{\text{Risk}}_{\text{class}=1,*}}{1-\widehat{\text{Risk}}_{\text{class}=1,*}}\right)\pm\frac{1.96}{\widehat{\text{Risk}}_{\text{class}=1,*}}\times\sqrt{\text{Var}\left(\log\widehat{\text{PR}}^{\text{profile}=1,0}\right)}\right]}{1+\exp\left[\log\left(\frac{\widehat{\text{Risk}}_{\text{class}=1,*}}{1-\widehat{\text{Risk}}_{\text{class}=1,*}}\right)\pm\frac{1.96}{\widehat{\text{Risk}}_{\text{class}=1,*}}\times\sqrt{\text{Var}\left(\log\widehat{\text{PR}}^{\text{profile}=1,0}\right)}\right]},$$

$$\frac{\exp\left[\log\left(\frac{\widehat{\text{Risk}}_{\text{class}=*,1}}{1-\widehat{\text{Risk}}_{\text{class}=*,1}}\right)\pm\frac{1.96}{\widehat{\text{Risk}}_{\text{class}=*,1}}\times\sqrt{\text{Var}\left(\log\widehat{\text{PR}}^{\text{profile}=0,1}\right)}\right]}{1+\exp\left[\log\left(\frac{\widehat{\text{Risk}}_{\text{class}=*,1}}{1-\widehat{\text{Risk}}_{\text{class}=*,1}}\right)\pm\frac{1.96}{\widehat{\text{Risk}}_{\text{class}=*,1}}\times\sqrt{\text{Var}\left(\log\widehat{\text{PR}}^{\text{profile}=0,1}\right)}\right]},$$

$$\frac{\exp\left[\log\left(\frac{\widehat{\text{Risk}}_{\text{class}=1,1}}{1-\widehat{\text{Risk}}_{\text{class}=1,1}}\right)\pm\frac{1.96}{\widehat{\text{Risk}}_{\text{class}=1,1}}\times\sqrt{\text{Var}\left(\log\widehat{\text{PRISM}}\right)}\right]}{1+\exp\left[\log\left(\frac{\widehat{\text{Risk}}_{\text{class}=1,1}}{1-\widehat{\text{Risk}}_{\text{class}=1,1}}\right)\pm\frac{1.96}{\widehat{\text{Risk}}_{\text{class}=1,1}}\times\sqrt{\text{Var}\left(\log\widehat{\text{PRISM}}\right)}\right]}.$$
