# Supplementary material for: Assessing Causal Mechanistic Interactions: A Peril Ratio Index of Synergy Based on Multiplicativity
Source: PLoS One. 2013 Jun 24;8(6):e67424. doi: 10.1371/journal.pone.0067424 (PMC3691192; doi:10.1371/journal.pone.0067424)
Supplement: Exhibit S7 — Synergy signal and follow-up time. (DOC) [file pone.0067424.s007.doc]

Supporting Information of

Assessing Causal Mechanistic Interactions: a Peril Ratio Index of Synergy based on Multiplicativity

Author: Wen-Chung Lee^1,2^

Author’s affiliation: 1. Research Center for Genes, Environment and Human Health,

College of Public Health, National Taiwan University, Taipei, Taiwan.

2. Institute of Epidemiology and Preventive Medicine,

College of Public Health, National Taiwan University, Taipei, Taiwan.

Correspondence & reprint requests: Prof. Wen-Chung Lee,

Rm. 536, No. 17, Xuzhou Rd., Taipei 100, Taiwan.

(FAX: 886-2-23511955)

(e-mail:wenchung@ntu.edu.tw)

Exhibit S7. Synergy signal and follow-up time.

Assuming proportional hazards and define the hazard ratio (HR) for people with $\text{profile}=x_{1},x_{2}$ as $\text{HR}^{\text{profile}=x_{1},x_{2}}=\frac{\text{Rate}^{\text{profile}=x_{1},x_{2}}(t)}{\text{Rate}^{\text{profile}=0,0}(t)},$ and the relative excess risk due to interaction in terms of hazard ratio, as $\text{RERI}_{\text{HR}}=\text{HR}^{\text{profile}=1,1}-\text{HR}^{\text{profile}=1,0}-\text{HR}^{\text{profile}=0,1}+1.$ Note that both the $\text{HR}^{\text{profile}=x_{1},x_{2}}$ and the $\text{RERI}_{\text{HR}}$ are time-invariant. Because a log peril is a cumulative rate [see Equation (1) in text], we have $\log\text{Peril}^{\text{profile}=x_{1},x_{2}}=\text{HR}^{\text{profile}=x_{1},x_{2}}\times\log\text{Peril}^{\text{profile}=0,0}=\text{HR}^{\text{profile}=x_{1},x_{2}}\times\log\left(1+\text{Odds}^{\text{profile}=0,0}\right).$ Taking logarithm on both sides of Equation (8) in text, we arrive at

$\log\text{PRISM}=\text{RERI}_{\text{HR}}\times\log\left(1+\text{Odds}^{\text{profile}=0,0}\right)\text{. }$ (S7.1)

$\text{Odds}^{\text{profile}=0,0}$ in (S7.1) increases monotonically as follow-up time $T$ increases. But this has no role whatsoever on the appearance/disappearance of a synergy signal: $\log\text{PRISM}=0$ ($\text{PRISM}=1$) if and only if $\text{RERI}_{\text{HR}}$ (a time-invariant constant) is zero in (S7.1).

If the disease odds for the four exposure profiles are approximately equal, the squared test statistic of the global (two-sided) PRISM test is approximately (S5)

$\begin{matrix} \text{E}\left(Z_{\text{global}}^{2}\right)=\text{E}\left[\frac{\log^{2}\widehat{\text{PRISM}}}{\text{Var}\left(\log\widehat{\text{PRISM}}\right)}\right] \\ \text{ }\approx\frac{\text{RERI}_{\text{HR}}^{2}}{\frac{1}{N_{11}}+\frac{1}{N_{10}}+\frac{1}{N_{01}}+\frac{1}{N_{00}}}\times\frac{\log^{2}\left(1+\text{Odds}^{\text{profile}=0,0}\right)}{\text{Odds}^{\text{profile}=0,0}}. \end{matrix}$ (S7.2)

$\text{E}\left(Z_{\text{global}}^{2}\right)$ in (S7.2) reaches a maximum when $\text{Odds}^{\text{profile}=0,0}\approx3.9216$ [$\frac{\log^{2}\left(1+x\right)}{x}$ has a maximum when $x\approx3.9216$], or $\text{Risk}^{\text{profile}=0,0}\approx0.7968$.
